# Supplementary material for: Genetic Signature of River Capture Imprinted in Schizopygopsis Fish from the Eastern Tibetan Plateau
Source: Genes (Basel). 2024 Aug 31;15(9):1148. doi: 10.3390/genes15091148 (PMC11431074; doi:10.3390/genes15091148)
Supplement: Supplementary file 1 [file genes-15-01148-s001.zip › Supporting Information.pdf]

Genetic signature of river capture imprinted in several *Schizopygopsis* fishes from the eastern Tibetan Plateau

SUPPORTING INFORMATION (SI)

Additional supporting information may be found online in the Supporting Information section.

References

1. Yang, D.Y., & Li, X.S. (2001) Study on the eastward flow of the Jinsha River. *J. Nanjing Univ. (Nat. Sci.)* **37**, 317-322.
2. Shi, Y.F., Li, J.J., Li, B.Y., Yao, T.D., Wang, S.M., Li, S.J., Cui, Z.J., Wang, F.B., Pan, B.T., Fang, X.M., & Zhang, Q.S. (1999) Uplift of the Qinghai-Xizang (Tibetan) Plateau and East Asia Environmental change during late Cenozoic. *Acta Geogr. Sin.* **54**, 10-20.
3. Tang, Y. T, Li, C. H., Wanghe, K. Y., Feng, C. G., Tong, C., Tian, F., & Zhao, K. (2019). Convergent evolution misled taxonomy in schizothoracine fishes (Cypriniformes: Cyprinidae). *Mol. Phylogenet. Evol.* **134**, 323-337.
4. Zhang, J. P., Liu, Z., Zhang, B., Yin, X.Y., Wang, L., Shi, H. N., & Kang, Y. J. (2015) Genetic diversity and taxonomic status of *Gymnocypris chilianensis* based on the mitochondrial DNA cytochrome b gene. Genet. Mol. Res. 14(3), 9253-9260.

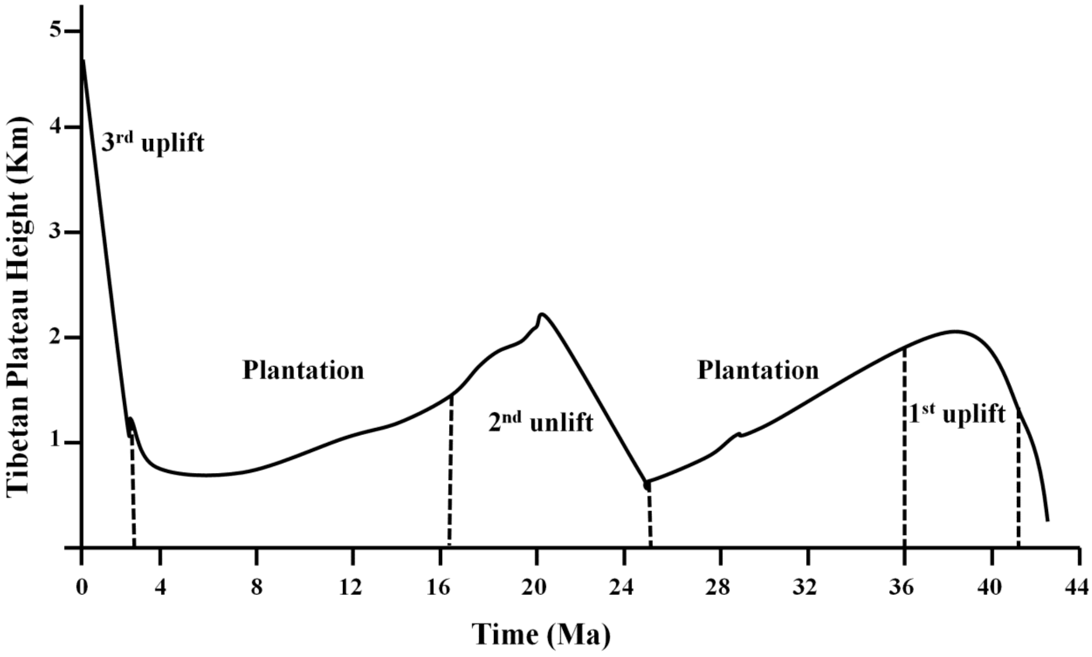

Figure S1. Schematic diagram of repeated uplifting and plantation on the Tibetan Plateau during Tertiary. Ma-Million Years Ago. Modified after Shi *et al*. (1999).

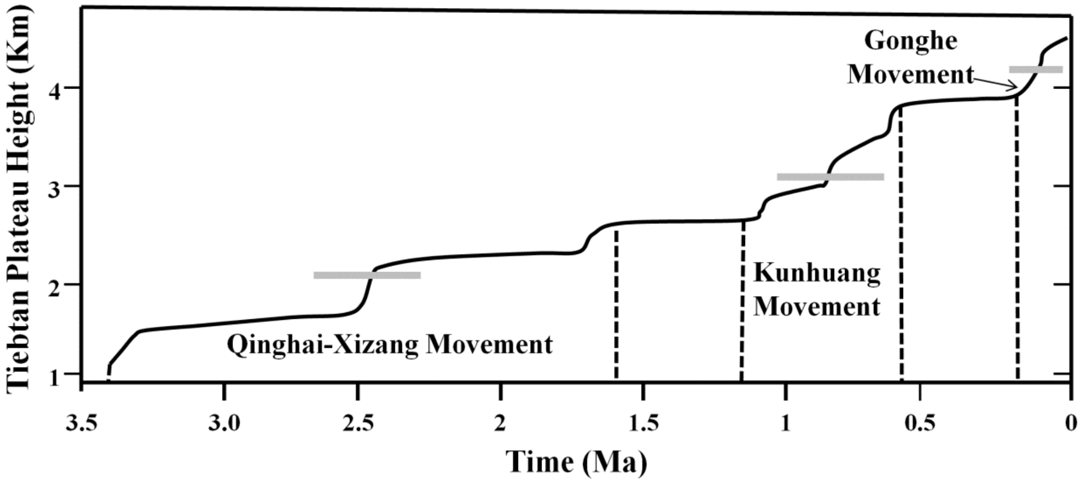

Figure S2. Uplifting process of Tibet Plateau since 3.4 Ma. Gray lines indicated the average Tibetan Plateau heights of three uplifting movements. Modified after Shi *et al*. (1999).

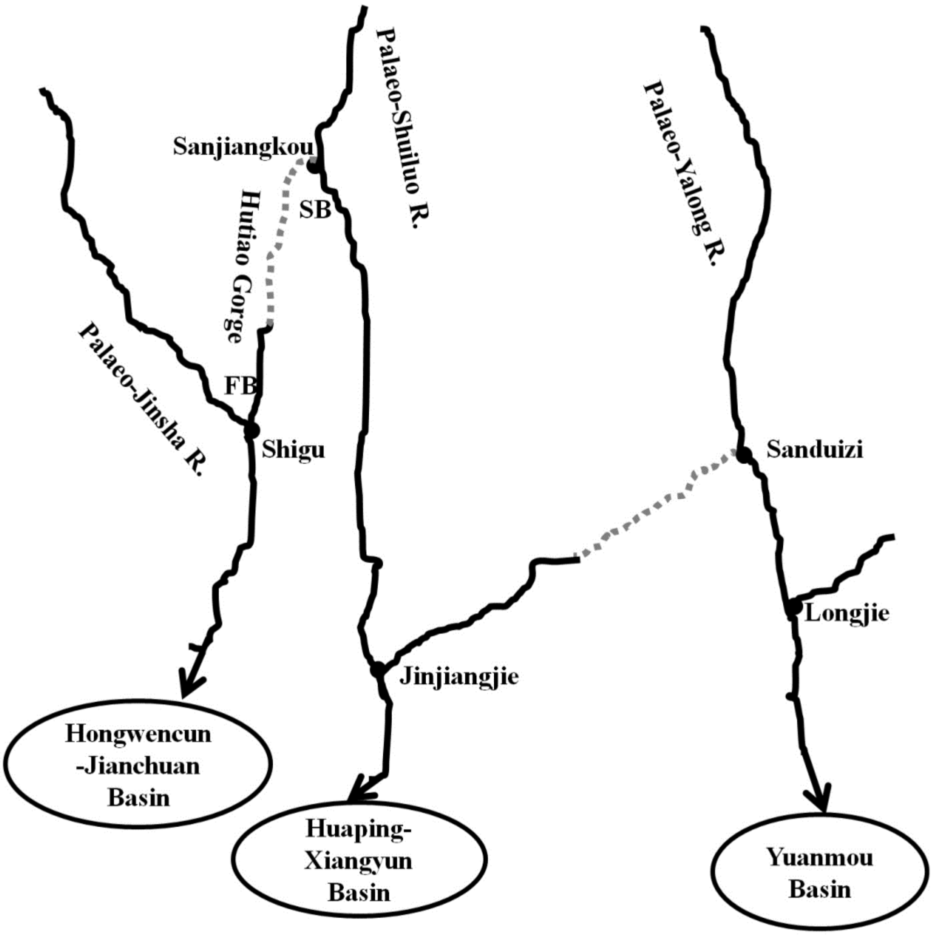

Figure S3. Parallel south flowing palaeo-rivers of the upper Changjiang/Yangtze system before middle Pleistocene. The palaeo-Jinsha, palaeo-Shuiluo and palaeo-Yalong rivers, three of which were believed to have emptied into three different basins. The grey dotted lines indicated contemporary connections and courses between them. R.-River. Modified after Yang & Li (2001).

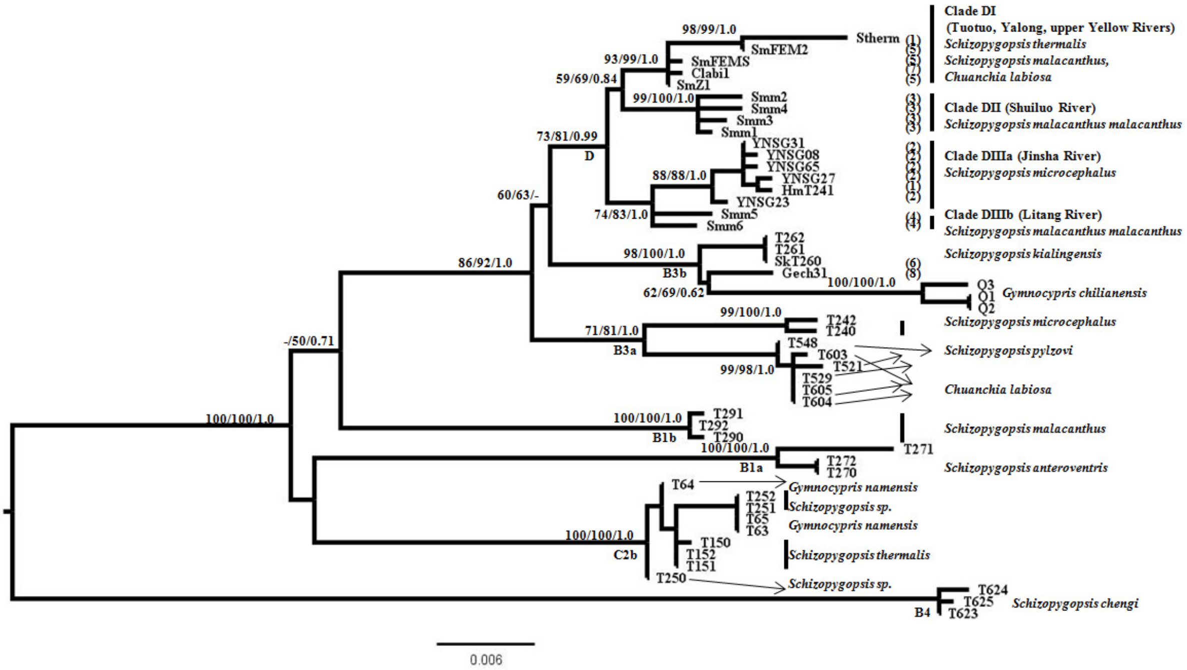

Figure S4 Phylogenetic tree of schizothoracine fishes based on 50 *Cyt b* gene sequences (Dataset I). Numbers above branches indicate nodal support values measured as bootstrap values (BP) and posterior probability (PP) inferred by Maximum Parsimony (MP), Maximum likelihood (ML) and Bayesian inference (BI) methodologies, respectively. Except new lineage D in this study, other lineages including B1a, B1b, B3 and B4 are same as those observed in Tang et al. (2019).

Table S1 Specimen information of 33 schizothoracine fish sequences from the Tibetan Plateau

Drainage (locality)	Species	Haplotype	GenBank number	Reference
Yellow River (Gande, Qinghai)	*Chuanchia labiosa*	T603	KY461371	Tang *et al*., 2019
Yellow River (Gande, Qinghai)	*Chuanchia labiosa*	T604	KY461372	Tang *et al*., 2019
Yellow River (Gande, Qinghai)	*Chuanchia labiosa*	T605	KY461373	Tang *et al*., 2019
Hexi Corridor, Heihe River (Gansu)	*Gymnocypris eckloni chilianensis*	Gech31	KM371149	Zhang *et al*., 2015
Hexi Corridor, Shiyang River (Wuwei, Gansu)	*Gymnocypris eckloni chilianensis*	Q1	KY461311	Tang *et al*., 2019
Hexi Corridor, Shiyang River (Wuwei, Gansu)	*Gymnocypris eckloni chilianensis*	Q2	KY461312	Tang *et al*., 2019
Hexi Corridor, Shiyang River (Wuwei, Gansu)	*Gymnocypris eckloni chilianensis*	Q3	KY461313	Tang *et al*., 2019
Lake Namtso (Damxung, Tibet)	*Gymnocypris namensis*	T63	KY461381	Tang *et al*., 2019
Lake Namtso (Damxung, Tibet)	*Gymnocypris namensis*	T64	KY461382	Tang *et al*., 2019
Lake Namtso (Damxung, Tibet)	*Gymnocypris namensis*	T65	KY461383	Tang *et al*., 2019
Lake Namtso (Damxung, Tibet)	*Gymnocypris namensis*	T250	KY461335	Tang *et al*., 2019
Lake Namtso (Damxung, Tibet)	*Gymnocypris namensis*	T251	KY461336	Tang *et al*., 2019
Lake Namtso (Damxung, Tibet)	*Gymnocypris namensis*	T252	KY461337	Tang *et al*., 2019
Yellow River (Gande, Qinghai)	*Schizopygopsis pylzovi*	T521	KY461363	Tang *et al*., 2019
Yellow River (Gande, Qinghai)	*Schizopygopsis pylzovi*	T529	KY461364	Tang *et al*., 2019
Yellow River (Gande, Qinghai)	*Schizopygopsis pylzovi*	T548	KY461367	Tang *et al*., 2019
Yangtze River, Jialing River (Têwo, Gansu)	*Schizopygopsis kialingensis*	SkT260	KY461338	Tang *et al*., 2019
Yangtze River, Jialing River (Têwo, Gansu)	*Schizopygopsis kialingensis*	T261	KY461339	Tang *et al*., 2019
Yangtze River, Jialing River (Têwo, Gansu)	*Schizopygopsis kialingensis*	T262	KY461340	Tang *et al*., 2019
Yangtze River, Jinsha River (Yushu, Qinghai)	*Schizopygopsis malacanthus*	T290	KY461344	Tang *et al*., 2019
Yangtze River, Jinsha River (Yushu, Qinghai)	*Schizopygopsis malacanthus*	T291	KY461345	Tang *et al*., 2019
Yangtze River, Jinsha River (Yushu, Qinghai)	*Schizopygopsis malacanthus*	T292	KY461346	Tang *et al*., 2019
Yangtze River, Tuotuo River (Germu, Qinghai)	*Schizopygopsis microcephalus*	T240	KY461331	Tang *et al*., 2019
Yangtze River, Tuotuo River (Germu, Qinghai)	*Schizopygopsis microcephalus*	T242	KY461333	Tang *et al*., 2019
Mekong River (Yushu, Qinghai)	*Schizopygopsis anteroventris*	T270	KY461341	Tang *et al*., 2019
Mekong River (Yushu, Qinghai)	*Schizopygopsis anteroventris*	T271	KY461342	Tang *et al*., 2019
Mekong River (Yushu, Qinghai)	*Schizopygopsis anteroventris*	T272	KY461343	Tang *et al*., 2019
Salween River, Naqu River (Cona, Tibet)	*Schizopygopsis thermalis*	T150	KY461318	Tang *et al*., 2019
Salween River, Naqu River (Cona, Tibet)	*Schizopygopsis thermalis*	T151	KY461319	Tang *et al*., 2019
Salween River, Naqu River (Cona, Tibet)	*Schizopygopsis thermalis*	T152	KY461320	Tang *et al*., 2019
Yangtze River, Dadu River (Banma, Qinghai)	*Schizopygopsis malacanthus chengi*	T623	KY461378	Tang *et al*., 2019
Yangtze River, Dadu River (Banma, Qinghai)	*Schizopygopsis malacanthus chengi*	T624	KY461379	Tang *et al*., 2019
Yangtze River, Dadu River (Banma, Qinghai)	*Schizopygopsis malacanthus chengi*	T625	KY461380	Tang *et al*., 2019
