# Supplementary figures and images for: Genetic Signature of River Capture Imprinted in Schizopygopsis Fish from the Eastern Tibetan Plateau
Source: Genes (Basel). 2024 Aug 31;15(9):1148. doi: 10.3390/genes15091148 (PMC11431074; doi:10.3390/genes15091148)

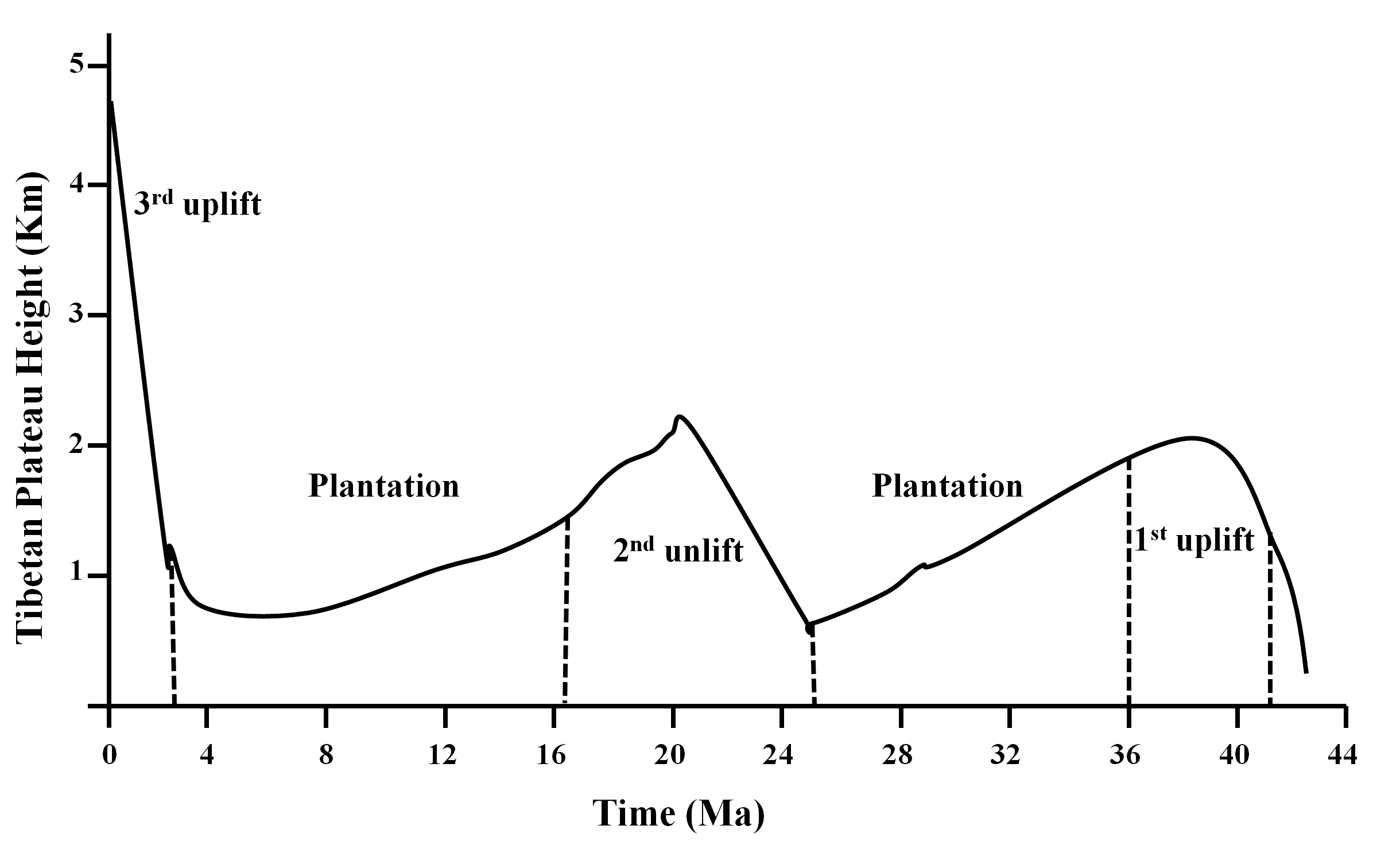

Supplement: Supplementary file 1 [file genes-15-01148-s001.zip › Figure S1.tif]

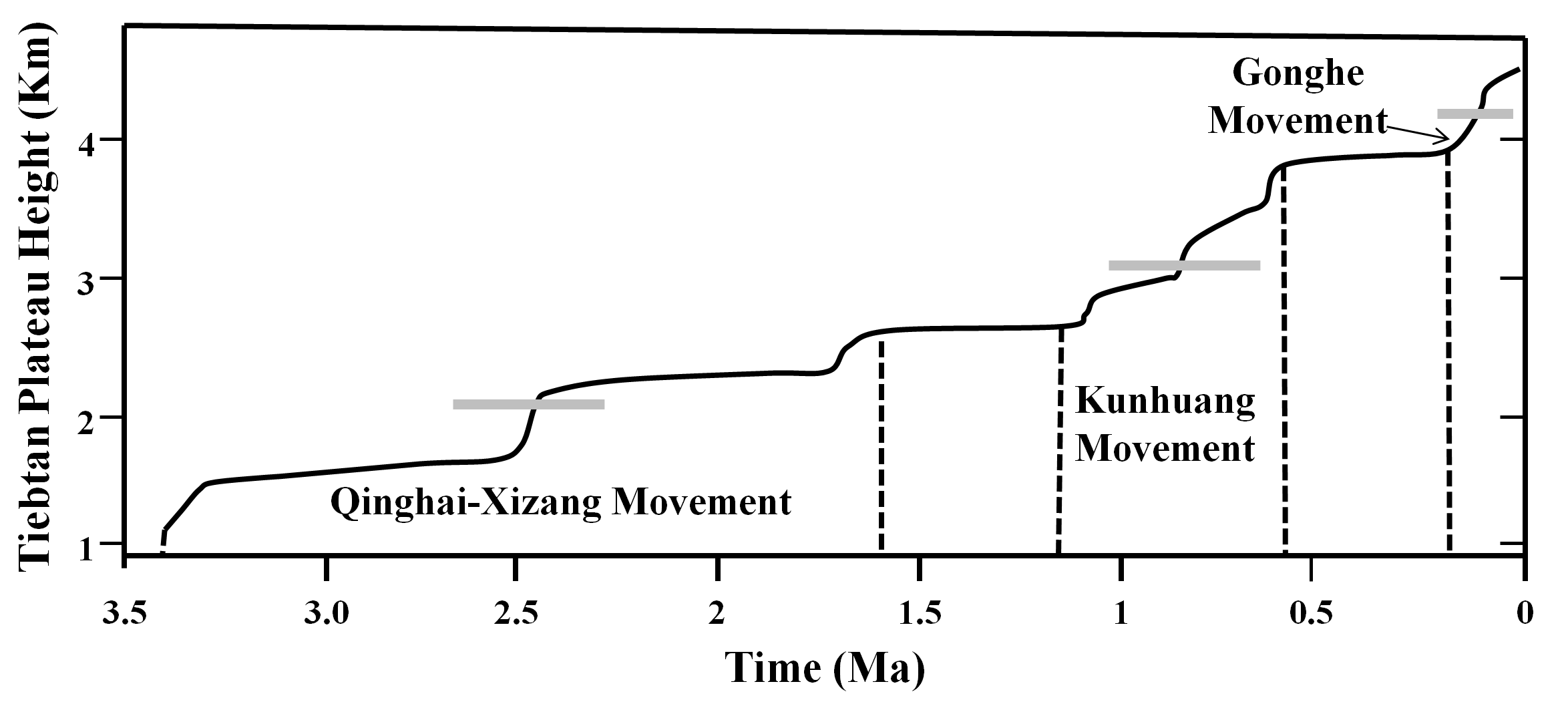

Supplement: Supplementary file 1 [file genes-15-01148-s001.zip › Figure S2.tif]

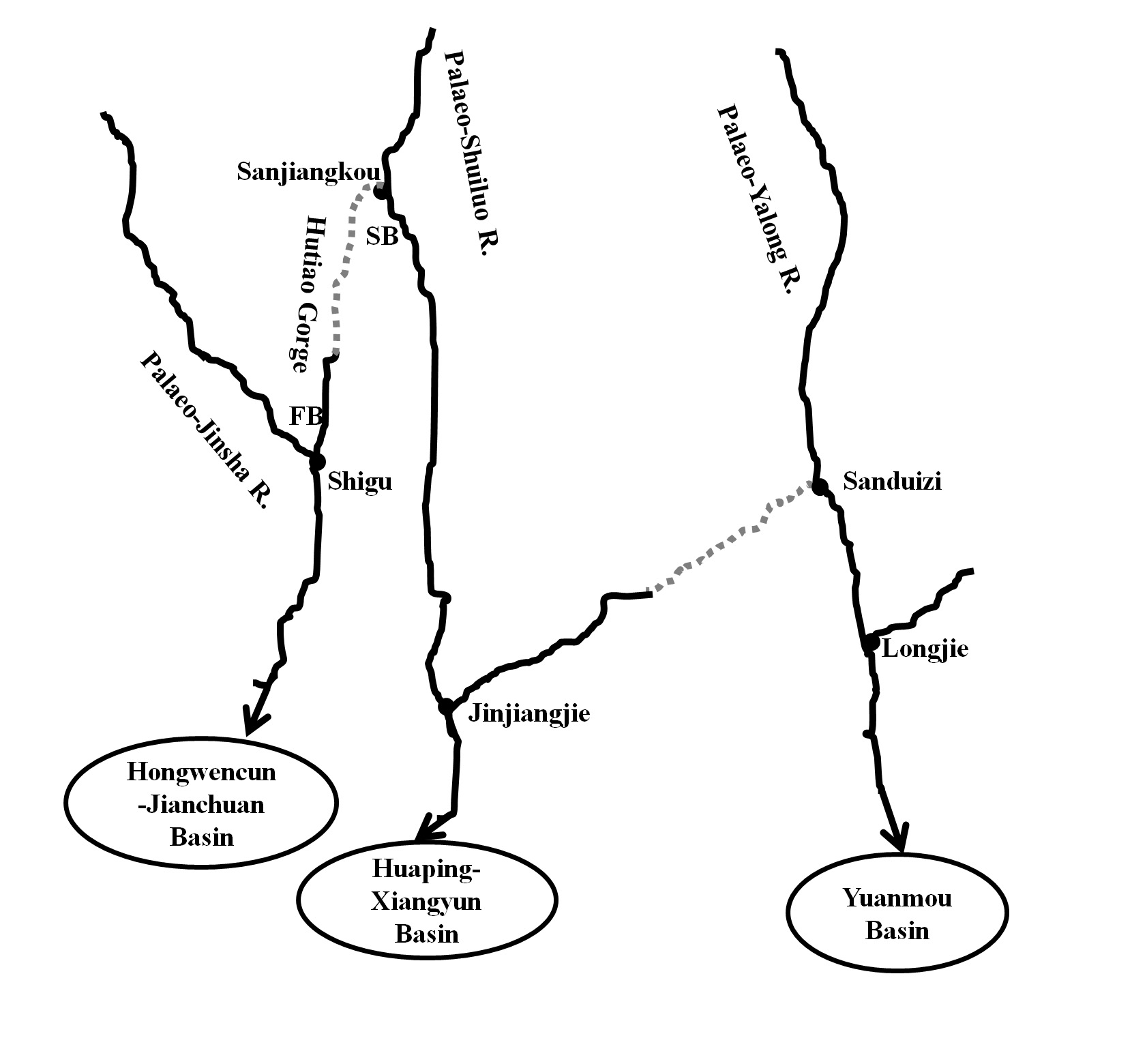

Supplement: Supplementary file 1 [file genes-15-01148-s001.zip › Figure S3.tiff]

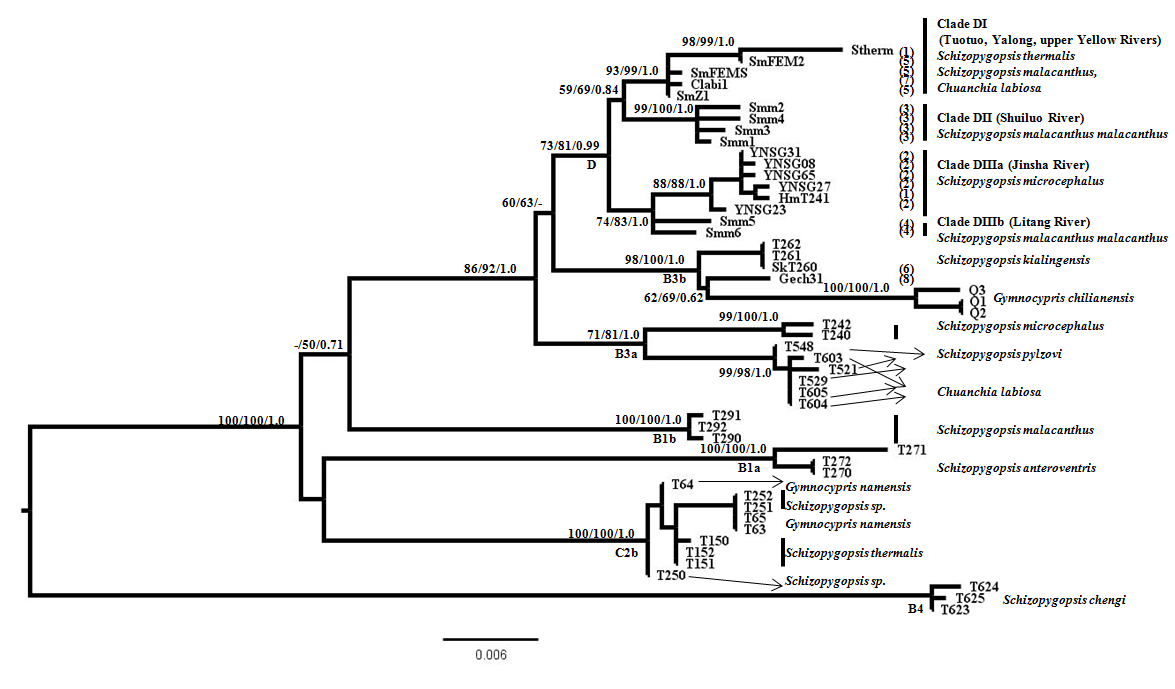

Supplement: Supplementary file 1 [file genes-15-01148-s001.zip › Figure S4.tif]
